# Dietary Chlorogenic Acid Attenuates Hepatic Lipid Accumulation and Reprograms Lipid Metabolism in Heat-Stressed Laying Hens: Integrated Transcriptomic and Metabolomic Analyses

**DOI:** 10.3390/biology15120917

**Published:** 2026-06-12

**Authors:** Dan Wang, Haiqiu Tan, Lin Peng, Xuanfu Wu, Jiang Gao, Wenqiang Ma

**Affiliations:** 1 Key Laboratory of Animal Physiology and Biochemistry, Ministry of Agriculture and Rural Affairs, College of Veterinary Medicine, Nanjing Agricultural University, Nanjing 210095, China; 2018207058@stu.njau.edu.cn (D.W.); 2024007036@stu.njau.edu.cn (H.T.); 2023007053@stu.njau.edu.cn (L.P.); 2023007052@stu.njau.edu.cn (X.W.); 2022007052@stu.njau.edu.cn (J.G.); 2 MOE Joint International Research Laboratory of Animal Health & Food Safety, Nanjing Agricultural University, Nanjing 210095, China

**Keywords:** chlorogenic acid, heat stress, peak laying period, fatty liver hemorrhagic syndrome, multi-omics, lipid metabolism

## Abstract

Heat stress leads to excessive hepatic lipid deposition and oxidative imbalance in laying hens, especially during peak laying period. Chlorogenic acid (CGA), a dietary polyphenol with antioxidant and lipid-modulating properties, may improve hepatic lipid homeostasis, yet its effects under heat-stress conditions remain unclear. In this study, 240 Hy-Line Brown laying hens at 36 weeks of age were randomly assigned to one of two treatments (120 hens per treatment, with six replicates of 20 hens each): a basal diet or a basal diet supplemented with 300 mg/kg CGA and subjected to heat-stress conditions for 8 weeks. CGA supplementation significantly reduced liver weight (25.3%), liver index (14.4%), hepatic triglyceride content (29.1%), and serum triglyceride level (61.7%) (*p* < 0.05). Histological assessment revealed lower steatosis and inflammation scores, alongside increased hepatic SOD activity (13.6%) and decreased MDA content (58.7%) (*p* < 0.05). RNA-seq analysis identified 420 differentially expressed genes that were significantly enriched in PPAR signaling and fatty acid β-oxidation pathways. CGA upregulated fatty acid oxidation-related genes (*ACSL1*, *CPT1A*, *ACOX1*, *ACAA1*) and downregulated lipogenic markers (*FASN*, *ACACA*). Serum metabolomics revealed coordinated changes in lipid and carbon metabolism. These results indicate that dietary CGA alleviates hepatic lipid accumulation and oxidative stress in heat-stressed peak-laying hens, potentially via PPARα-mediated enhancement of fatty acid oxidation and inhibition of de novo lipogenesis.

## Simple Summary

Heat stress causes oxidative damage and fat accumulation in the liver of laying hens, reducing their health and productivity. This study investigated whether chlorogenic acid, a natural plant compound found in many foods, could protect the liver of peak-laying hens exposed to high ambient temperatures. Hens receiving chlorogenic acid in their diet for eight weeks showed substantially lower liver fat content and reduced liver inflammation, alongside improved antioxidant capacity. Analysis of gene expression and blood metabolites revealed that chlorogenic acid promoted fat breakdown while suppressing fat synthesis in the liver. These findings suggest that adding chlorogenic acid to poultry feed is a practical and effective nutritional strategy for maintaining liver health in laying hens during hot weather conditions.

## 1. Introduction

Modern laying hens, typically managed within highly intensive production systems, have been selectively bred for superior egg output [1,2,3]. Although these advances have markedly improved production efficiency, they concurrently impose a pronounced metabolic burden, particularly during the peak laying period (30–40 weeks of age), when daily egg production rates exceed 90%, and hepatic lipid flux reaches its physiological maximum [4,5,6]. Unlike mammals, avian species rely almost exclusively on hepatic tissue for endogenous lipid synthesis (de novo lipogenesis) and the assembly of yolk lipoproteins, rendering the liver uniquely susceptible to lipid overload [7,8]. During this peak period, the concurrent demands of maximizing vitellogenesis and sustaining basal metabolism create a state of heightened hepatic vulnerability: hepatic lipogenic gene expression peaks, antioxidant reserves are partially depleted, and the capacity for very-low-density lipoprotein (VLDL) export may be outpaced by fatty acid influx [1,9,10]. Any perturbation to this precarious balance predisposes birds to ectopic fat deposition and the clinical sequelae collectively described as fatty liver hemorrhagic syndrome (FLHS), a major noninfectious metabolic disorder in commercial layers associated with impaired health, reduced productive lifespan, and significant economic losses [9,11,12,13].

Thermal stress represents one of the most economically detrimental environmental constraints facing the modern poultry industry [14,15]. The thermoneutral zone for laying hens generally spans 18–24 °C; sustained exposure above approximately 28 °C constitutes chronic heat stress and triggers cascading adverse physiological responses [16,17]. Elevated temperature–humidity indices suppress feed intake, dysregulate the hypothalamic–pituitary–adrenal (HPA) axis, impair intestinal barrier integrity, and disrupt redox homeostasis [18,19]. Under heat stress, excessive reactive oxygen species (ROS) production and amplified pro-inflammatory signaling exacerbate hepatic lipid accumulation by simultaneously upregulating de novo lipogenesis and suppressing β-oxidation, thereby aggravating the predisposition to FLHS [20,21]. Given that laying hens synthesize the vast majority of fatty acids within the liver, targeted nutritional interventions aimed at mitigating hepatic lipogenesis or enhancing fatty acid oxidation represent practical and effective strategies to prevent hepatic lipid overload during periods of environmental stress.

As policies increasingly restrict and ban the use of antimicrobials, optimizing feed nutrition has emerged as an effective strategy for preventing and alleviating lipid disorders in laying hens [22]. Chlorogenic acid (CGA) is a naturally occurring polyphenol widely present in coffee, tea, fruits, and vegetables and has been well documented for its broad-spectrum biological activities, including antioxidant, anti-inflammatory, antimicrobial, and immunomodulatory properties [23,24,25,26]. Mechanistically, CGA and its metabolites—particularly caffeic acid and ferulic acid generated by intestinal microbiota—have been shown to activate peroxisome proliferator-activated receptor α (PPARα), a master transcriptional regulator of hepatic fatty acid oxidation, in both in vitro hepatocyte models and in vivo rodent studies [27,28,29]. However, avian-specific evidence for these mechanisms remains comparatively limited. In laying hens, PPARα activation drives mitochondrial and peroxisomal β-oxidation of the long-chain fatty acids destined for yolk deposition, and its upregulation has been associated with reduced hepatic triglyceride accumulation in nutritional challenge models [30,31,32]. Recent studies in poultry have further demonstrated that CGA or CGA-rich plant extracts improve antioxidant capacity, intestinal barrier function, and lipid metabolism indices, and CGA supplementation may reduce hepatic lipid accumulation and improve liver function in aged laying hens [32]. Nevertheless, direct evidence linking CGA to PPARα-mediated fatty acid oxidation in the avian liver under heat-stress conditions has not been established.

Most previous studies have focused on hens in the late laying period (>60 weeks), while investigations during the peak laying period remain limited. As noted above, this stage is characterized by maximal lipogenic pressure and elevated oxidative burden, rendering hepatic lipid homeostasis particularly fragile; dysregulation during this window may compromise hepatic health across subsequent laying cycles. Furthermore, the integrated hepatic transcriptomic and circulating metabolomic responses to CGA under heat-stress conditions have not been characterized in any avian model. We hypothesized that dietary CGA supplementation would attenuate hepatic lipid accumulation and mitigate FLHS-associated pathology in heat-stressed peak-laying hens by enhancing PPARα-driven fatty acid oxidation and suppressing de novo lipogenesis. Therefore, this study integrated hepatic histology, biochemical assays, RNA-seq transcriptomics, serum untargeted metabolomics, qRT-PCR, and Western blotting to examine the effects of dietary CGA supplementation on hepatic lipid metabolism in heat-stressed peak-laying hens.

## 2. Materials and Methods

### 2.1. Animal Experiment, Management, and Diets

The animal protocol for this study received approval from the Institutional Animal Care and Use Committee of Nanjing Agricultural University (No: NJAULLSC2023056). Two hundred and forty Hy-Line Brown laying hens at 36 weeks of age were randomly assigned to one of two experimental treatments based on their initial body weight and egg production rate, with each treatment comprising six independent replicates of twenty hens. The control group (CON) was fed a basal diet, while the CGA group was provided with a diet formulated by supplementing the basal diet with 300 mg/kg of CGA. The inclusion level of 300 mg/kg CGA was determined based on previously published poultry studies [33,34,35] and unpublished preliminary data from our laboratory. The CGA was premixed with a small amount of basal diet and then thoroughly mixed into the total diet to ensure homogeneity. The CGA (purity > 98%) used in this study was derived from honeysuckle extract and purchased from Changsha Shanghe Biotechnology Co., Ltd. (Changsha, China). Hens were housed in conventional three-tier battery cages (50 cm × 45 cm × 45 cm, length × depth × height), with five hens per cage and four cages per replicate, providing a stocking density of 450 cm^2^ per hen. Replicate cages from both treatment groups were distributed across all three tiers and arranged in an interspersed pattern throughout the house to minimize potential positional bias arising from the tier height, ventilation, and lighting gradients. The experimental period consisted of a total of 9 weeks, including one week adaptation and an 8-week formal experimental phase. Throughout the entire experimental period, the animals were allowed ad libitum access to feed and water. The photoperiod was held constant at 16 h light and 8 h dark for the duration of the study.

The experiment was conducted during July and August, taking advantage of naturally high ambient temperatures to induce heat stress. To maintain heat stress conditions throughout the trial, artificial interventions were implemented, including restriction of ventilation and closure of the evaporative cooling pads (wet curtains) in the house. The temperature and relative humidity were monitored continuously throughout the experimental period. The recorded temperature ranged from 24.35 °C to 28.21 °C (mean ± SD: 26.54 ± 0.96 °C), and the relative humidity ranged from 81.85% to 95.37% (mean ± SD: 92.25 ± 3.44%), resulting in a temperature–humidity index (THI) consistently ≥73, which exceeds the established comfort threshold for laying hens [36,37]. The composition and nutrient content of the basal diet was formulated according to the GB/T 5916-2020 [38] recommendations (Table 1).

### 2.2. Measurements of Laying Performance and Egg Quality

The egg number and weight were recorded daily for each replicate. The feed intake was recorded weekly for each replicate. These data were used to calculate the average egg weight and feed-to-egg ratio. At the end of the 4th and 8th weeks, 5 eggs were randomly collected from each replicate, with 30 eggs in total per group. The egg quality parameters, including the egg weight, albumen height, yolk color, and Haugh unit, were determined using the EMT-5200 Multi-functional Egg Quality Analyzer (Robotmation Co., Ltd., Tokyo, Japan). The eggshell weight and yolk weight were measured using an electronic balance (SF-400, BAIJIE, Huzhou, China). The albumen weight was calculated by subtracting the yolk weight and eggshell weight from the whole egg weight. The eggshell strength was evaluated using an EFR-01 eggshell strength tester (ORKA Food Technology Ltd., Herzeliya, IL, USA). The shell thickness at the blunt end, sharp end, and equatorial region without the inner membrane was measured using a spiral micrometer, and the average shell thickness was calculated.

### 2.3. Sample Collection

The final body weights of laying hens were recorded before sample collection. Upon completion of the eight-week dietary intervention, the hens were fasted overnight for approximately 12 h; thereafter, two individuals per replicate were randomly chosen, and venipuncture of the brachial vein was performed to collect blood samples. The blood was centrifuged at 4 °C, and the separated serum was stored at −80 °C until further analysis. The hens were then sacrificed by exsanguination following cervical dislocation. Liver tissues were immediately excised, weighed, and photographed to observe the morphological characteristics. The liver index = liver weight (g)/body weight (g) × 100%. Subsequently, liver samples from the same anatomical location were rapidly collected, snap-frozen in liquid nitrogen, and stored at −80 °C for subsequent analyses. Additionally, liver tissue samples of approximately 0.5 cm × 0.5 cm × 0.5 cm were fixed in 4% paraformaldehyde solution for histopathological section preparation. For downstream analyses, subsets of birds were independently and randomly selected from each group. The same six birds per group were used for both hepatic transcriptomics (RNA-seq) and serum untargeted metabolomics to enable integrated multi-omics correlation analyses. Four birds per group were randomly selected for histological examinations (H&E and Oil Red O staining). Six birds per group were randomly selected for qPCR, Western blot, and biochemical analyses.

### 2.4. Hepatic Histological Assessment

Hepatic specimens were immersed in neutral-buffered formalin at 4% concentration for no less than 24 h to achieve adequate fixation, followed by serial dehydration through graded alcohols and clearing in xylene. These transparent samples were embedded in paraffin, cut into 5-μm slices, and stained with hematoxylin and eosin (H&E; Solarbio Science & Technology Co., Ltd., Beijing, China) to observe pathological changes. Steatosis and inflammation were scored by two independent treatment-blinded investigators according to previous methods [39,40]. Steatosis was graded on a scale of 0 to 3 (0: absent; 1: mild; 2: moderate; 3: severe) based on the extent of lipid accumulation in hepatocytes. The inflammatory severity was similarly scored from 0 to 3 according to the degree of inflammatory cell infiltration. At least five non-overlapping fields per liver section were examined. For Oil Red O staining, the liver tissues were directly embedded at the optimal cutting temperature (OCT) and rapidly frozen at −80 °C for 5–10 min. Sections were cut using a cryostat microtome (Thermo Fisher Scientific, Waltham, MA, USA), with a thickness of 4–6 μm (sections too thick are prone to detachment, while those too thin affect observation). The sections were stained with ORO and counterstained with hematoxylin. All sections were scanned using a Pannoramic 250 Flash III slide scanner (3DHistech, Ltd., Budapest, Hungary), and images were acquired using Case-Viewer software (version 2.3, 3DHistech, Ltd., Budapest, Hungary).

### 2.5. Determination of Biochemical Parameters in Liver and Serum

Serum and hepatic triglycerides (TG), total cholesterol (TC) and free cholesterol (FC) were measured using the corresponding kits (Applygen Technologies, Beijing, China), according to the manufacturer’s instructions. The cholesterol esters (CE) content was determined by subtracting FC from TC and expressed as cholesterol equivalents. The hepatic antioxidant status was assessed by measuring superoxide dis-mutase (SOD), glutathione peroxidase (GSH-PX), and catalase (CAT) activities, as well as glutathione (GSH) and malondialdehyde (MDA) contents. All hepatic biochemical data were normalized to the total protein concentration determined by the BCA method. The assay kits were supplied by Nanjing Jiancheng Bioengineering Institute (Nanjing, China).

### 2.6. Hepatic Transcriptome Sequencing and Enrichment Analysis

Twelve liver samples (six per group) were utilized for transcriptomic analysis. Total RNA was extracted from livers using the Trizol method. RNA quality was assessed by OD260/OD280 and OD260/OD230 ratios, and RNA degradation and contamination were evaluated by 1% agarose gel electrophoresis. Subsequently, cDNA libraries were constructed and sequenced on the Illumina NovaSeq platform (Illumina, Inc., San Diego, CA, USA) by Shanghai Personalbio Technology Co., Ltd. (Shanghai, China). Raw reads were subjected to quality filtering, and the resulting high-quality clean reads were aligned to the chicken reference genome (Gallus gallus; GCF_016699485.2; https://www.ncbi.nlm.nih.gov/datasets/genome/GCF_016699485.2/ (accessed on 5 December 2025)) using HISAT2 (v2.2.1). Gene expression levels were then quantified based on the alignment results. Principal component analysis (PCA) was performed on all samples based on gene expression profiles to assess inter-sample relationships and within-group reproducibility, with higher proximity between samples indicating higher transcriptomic similarity. Differentially expressed genes (DEGs) were identified using DESeq2 with the following criteria: |log2FC| > 1 and FDR-adjusted *p*-value < 0.05.

Subsequently, Kyoto Encyclopedia of Genes and Genomes (KEGG) and Gene Ontology (GO) annotations of the DEGs were obtained from the reference genome, and enrichment analysis was conducted using clusterProfiler 4.0 (https://bioconductor.org/packages/release/bioc/html/clusterProfiler.html (accessed on 5 December 2025)). Gene Set Enrichment Analysis (GSEA) (https://www.gsea-msigdb.org/gsea/index.jsp (accessed on 5 December 2025)) can effectively address issues such as the insufficient mining of effective information from minor-effect genes in traditional enrichment analysis. It provides a more comprehensive interpretation of the regulatory role of a functional unit and can complement traditional enrichment analysis. The *p*-values were computed using the hypergeometric distribution method, with an enrichment criterion of *p* < 0.05.

### 2.7. Serum Non-Targeted Metabolomic Analysis

Serum samples (200 µL each, *n* = 6) were mixed with 400 µL methanol, vortexed, and centrifuged to obtain the supernatant. The supernatant was vacuum-dried and reconstituted in 80% methanol containing 2-chlorophenylalanine as the internal standard. Quality control (QC) samples were prepared by pooling equal aliquots from all individual samples and were interspersed throughout the analytical run (every 6–12 samples) to monitor instrument stability and analytical reproducibility [41]. Prior to sample injection, 2–4 QC samples were injected for system equilibration. LC-MS analysis was performed using a Thermo Vanquish Flex UHPLC system coupled to a Thermo Orbitrap Exploris 120 mass spectrometer (Thermo Fisher Scientific, Waltham, MA, USA), controlled by Xcalibur software (version 4.7). Chromatographic separation was achieved on an ACQUITY UPLC HSS T3 column (100 Å, 1.8 µm, 2.1 mm × 100 mm; Waters Corporation, Milford, MA, USA) maintained at 40 °C, with an autosampler temperature of 8 °C and an injection volume of 2 µL. The mobile phase consisted of (A) 0.1% formic acid in water and (B) acetonitrile containing 0.1% formic acid, delivered at a flow rate of 0.4 mL/min. Gradient elution was programmed as follows: 0–1 min, 5% B; 1–4.7 min, 5–95% B; 4.7–6 min, 95% B; 6–6.1 min, 95–5% B; 6.1–8.5 min, 5% B. Mass spectrometric data were acquired in both positive and negative ionization modes using data-dependent acquisition (DDA). Ionization was performed with a heated electrospray ionization (HESI) source with spray voltages of +3.5 kV (positive) and −3.0 kV (negative), sheath gas at 40 arb, auxiliary gas at 10 arb, capillary temperature at 320 °C, and auxiliary gas temperature at 300 °C. Full-scan MS1 spectra were acquired over a mass range of *m/z* 70–1000 at a resolution of 60,000 (AGC Target: Standard; Max IT: 100 ms). The top 4 precursor ions were selected for HCD fragmentation (collision energy: 30%) with a dynamic exclusion time of 4 s; MS2 spectra were acquired at a resolution of 15,000 (AGC Target: Standard; Max IT: Auto).

Raw data were imported into MS-DIAL (version 4.9.221218) for peak detection, alignment, and filtering. Features detected in fewer than 50% of the QC samples were removed, and missing values for the remaining features were imputed using the gap-filling algorithm implemented in the software, followed by normalization. The relative standard deviation (RSD) of peak intensities across QC samples was calculated to assess the analytical reproducibility, and features with RSD > 30% were excluded. Metabolite identification was performed by matching against multiple databases, including the self-built PSNGM library, mzCloud, LIPID MAPS, HMDB, MoNA, and the NIST 2020 MS/MS spectral library. Key identification parameters were set as follows: MS1 tolerance, 0.01 Da; MS2 tolerance, 0.05 Da; minimum peak height, 10,000; identification score cut-off, 70. Identification confidence levels were assigned according to the minimum reporting standards of the Metabolomics Standards Initiative Chemical Analysis Working Group [42,43].

Multivariate statistical analysis was conducted using orthogonal partial least-squares discriminant analysis (OPLS-DA) to visualize group separation. The model validity was assessed by R^2^Y, Q^2^ values, cross-validation, and permutation tests. Differentially abundant metabolites (DAMs) were identified based on variable importance in projection (VIP) > 1 and Student’s *t*-test *p* < 0.05. KEGG pathway enrichment analysis of the DAMs was performed using clusterProfiler (v4.6.0).

### 2.8. RNA Extraction, cDNA Synthesis, and Quantitative Real-Time PCR (qRT-PCR)

Six hens per group were selected for qRT-PCR analysis. Hepatic total RNA was isolated with a guanidinium thiocyanate–phenol–chloroform reagent (Thermo Fisher Scientific, Waltham, MA, USA), and the yield together with the purity were assessed using a NanoDrop spectrophotometer (Thermo Scientific, Ottawa, ON, Canada) prior to downstream processing. The RNA samples were then diluted to a final concentration of 0.5 μg/μL with diethylpyrocarbonate (DEPC)-treated water (Biosharp, Beijing, China). cDNA synthesis was performed according to the manufacturer’s instructions (Vazyme Biotech Co., Ltd., Nanjing, China) with a 15 min reverse transcription at 37 °C, followed by a 5 s termination at 85 °C. Quantitative real-time PCR was conducted using a SYBR Green PCR kit (Vazyme Biotech Co., Ltd., Nanjing, China) on a CFX Connect Real-Time PCR Detection System (Bio-Rad Laboratories, Hercules, CA, USA). The PCR program consisted of initial denaturation at 95 °C for 30 s, followed by 40 cycles of 95 °C for 10 s and 60 °C for 30 s. Dissociation curve analysis was performed after each PCR run to confirm the specificity of the amplified products. Gene expression levels were normalized to the housekeeping gene β-Actin and calculated using the 2^−ΔΔCt^ method. Primer sequences for all target genes examined in this study are listed in Table 2.

### 2.9. Western Blot

Six hens per group were used for Western blot analysis. Hepatic protein extraction was carried out by mechanical homogenization of frozen liver fragments in ice-cold lysis buffer supplemented with both phosphatase and protease inhibitor cocktails, following a previously established protocol [44]. The protein concentration in the supernatant after centrifugation was determined using a BCA assay kit (Nanjing Jiancheng Bioengineering Institute, Nanjing, China). Protein separation was achieved by denaturing 10% polyacrylamide gel electrophoresis with 40 μg of total protein loaded per well, after which proteins were electrotransferred onto nitrocellulose membranes under constant current conditions. Membranes were blocked with 5% non-fat dry milk in TBST for 1.5 h at room temperature before primary antibody incubation. Briefly, the membranes containing protein were incubated with the primary anti-PPARα (1:1000), anti-ACSL1 (1:1000), anti-CPT1A (1:1000), anti-ACAA1 (1:1000), anti-ACOX1 (1:1000), anti-ACACA (1:1000) and anti-FASN (1:1000) antibodies at 4 °C overnight. After incubating with the horseradish peroxidase (HRP)-labeled goat anti-rabbit IgG (1:5000), the proteins were determined using the ultra-sensitive ECL chemiluminescence kit (RM00020P, ABclonal, Wuhan, China) by the digital imaging equipment (GE Healthcare, Chicago, IL, USA). α-Tubulin (1:10000) was used as the loading control, and all antibodies were purchased from ABclonal Biotechnology Co., Ltd. (Wuhan, China). Images were quantified by Image J 1.53k software (NIH, Bethesda, MD, USA).

### 2.10. Statistical Analysis

All data were tested for normality using the Shapiro–Wilk test and for homogeneity of variance using Levene’s test prior to parametric analyses. For normally distributed data, Student’s *t*-test was applied for two-group comparisons. Non-normally distributed data were analyzed using the Mann–Whitney U test. For transcriptomic and metabolomic datasets, FDR correction was applied using the Benjamini–Hochberg procedure to account for the large number of simultaneous statistical tests inherent to high-throughput profiling, with FDR-adjusted *p*-values used as the significance threshold. Data are expressed as mean ± standard error of the mean (SEM). All analyses were performed using SPSS 27.0 (IBM Corp., Armonk, NY, USA) and R version 4.3.0. Graphs were created using GraphPad Prism 10 (GraphPad Software, San Diego, CA, USA).

## 3. Results

### 3.1. Laying Performance and Egg Quality

**Figure 1:**
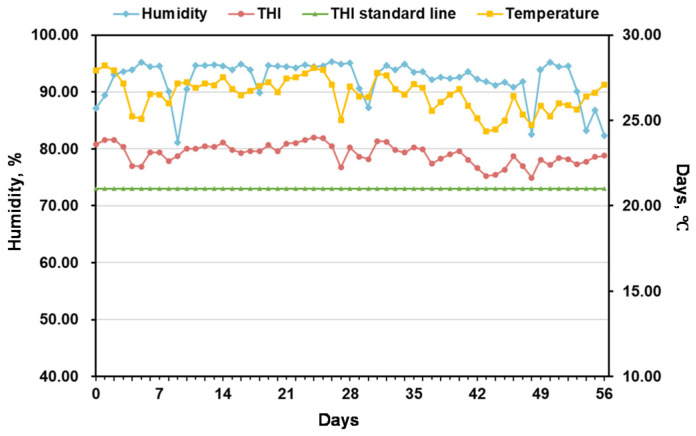
Temperature and humidity index (THI) curve.

During the 8-week experiment, the THI index remained above 73, confirming the presence of heat stress (Figure 1). Dietary CGA had no detectable influence on the laying rate, average egg weight, or feed efficiency (*p* > 0.05, Table 3). At the end of week 4, the albumen height and Haugh unit were enhanced in the CGA group, while the eggshell weight was significantly lower than that in the CON group (*p* < 0.05, Table 4). At the end of week 8, the yolk color score of the CGA group was significantly higher than that in CON group (*p* < 0.05, Table 4).

### 3.2. Effects of CGA on Hepatic Health

**Figure 2:**
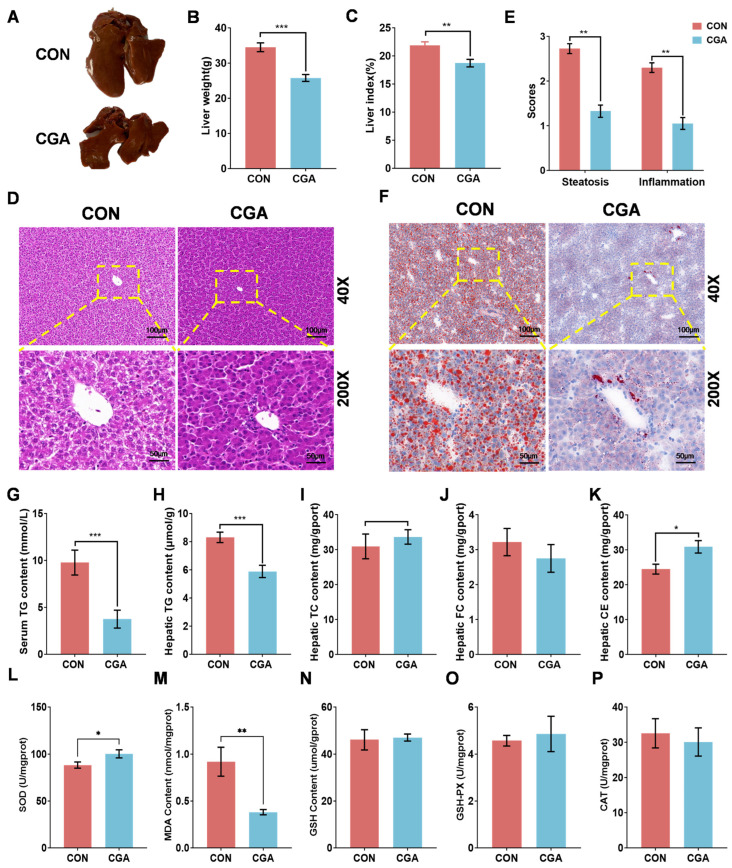
Effects of CGA on hepatic health. (**A**) Typical macroscopic morphology. (**B**) Liver weight (*n* = 12). (**C**) Liver index (*n* = 12). (**D**) Representative H&E staining images (*n* = 4). (**E**) Scoring of steatosis and inflammation in H&E sections. Data are represented as mean ± SEM (*n* = 4). (**F**) Representative Oil Red O staining images (*n* = 4). Serum (**G**) and hepatic (**H**) TG content. Hepatic TC (**I**), FC (**J**), and CE (**K**) content. Oxidative stress indicators SOD (**L**), MDA (**M**), GSH (**N**), GSH-PX (**O**), and CAT (**P**) levels. Data are represented as mean ± SEM (*n* = 6) *, *p* < 0.05; **, *p* < 0.01; ***, *p* < 0.001.

CGA supplementation significantly ameliorated the yellowish appearance of the liver (Figure 2A) and reduced both the liver weight and liver index compared with the control group (*p* < 0.05, Figure 2B,C). H&E staining sections of the liver from laying hens fed a CGA-supplemented diet exhibited fewer and smaller lipid droplets compared with the control group (Figure 2D). The steatosis and inflammation scores were significantly reduced compared with the control group (*p* < 0.05, Figure 2E). Oil Red O staining sections revealed a significant reduction in red-stained areas in the CGA group (Figure 2F). This finding was corroborated by the decreased levels of TG in both the liver and serum (Figure 2G,H). Supplementation of CGA in the diet had no effect on the levels of TC and FC, but it increased the content of CE in the liver (*p* < 0.05, Figure 2I–K). CGA supplementation increased hepatic SOD activity and decreased the MDA content (*p* < 0.05; Figure 2L,M), while GSH, GSH-PX and CAT did not differ significantly between groups (Figure 2N–P).

### 3.3. Hepatic Transcriptomic Changes

**Figure 3:**
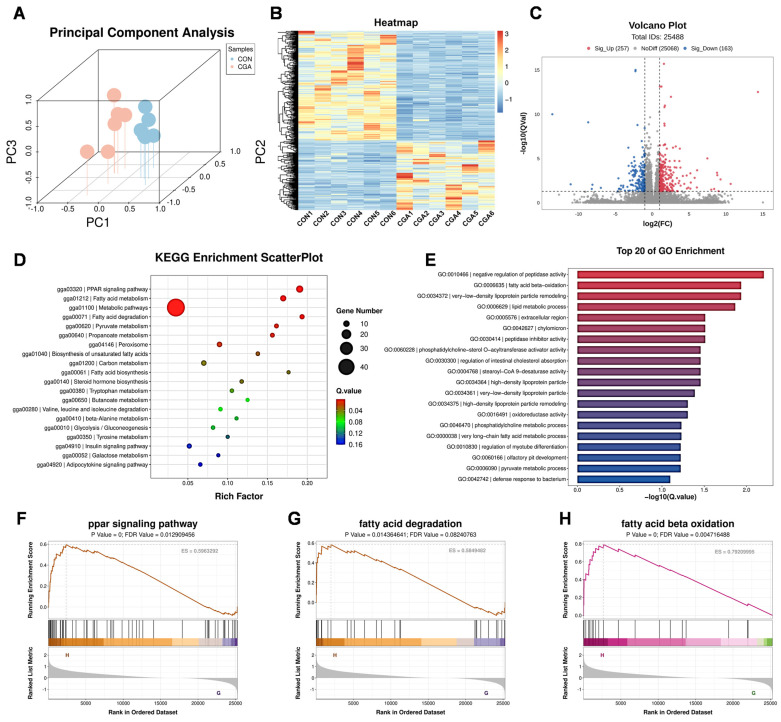
Effects of CGA on hepatic transcriptional profile. (**A**) 3D PCA plot. (**B**) DEG heatmaps. (**C**) Volcanic plot. Top 20 KEGG (**D**) and GO (**E**) enrichment pathways. GSEA enrichment analysis in PPAR signaling pathway (**F**), fatty acid degradation (**G**), and fatty acid beta oxidation (**H**).

RNA-Seq analysis was performed on liver samples from the CON and CGA groups. PCA was conducted based on gene expression levels. The results demonstrated that samples within each group were closely clustered, indicating high reproducibility of the samples, and substantial intergroup differences were observed between the CON and CGA groups, making them suitable for subsequent in-depth analysis (Figure 3A). The similarity and heterogeneity of samples from each group are shown as a heatmap (Figure 3B). The sequencing analysis result indicated that there was a total of 420 DEGs, screening out 257 upregulated genes and 163 downregulated genes, visualized as volcano plot (Figure 3C). To better understand the changes in liver gene expression caused by dietary CGA, KEGG and GO enrichment analysis was done on DEGs. KEGG pathway analysis revealed that the DEGs were significantly enriched in Peroxisome Proliferator-Activated Receptor (PPAR) signaling pathway, fatty acid metabolism, and fatty acid degradation (Figure 3D). GO enrichment analysis further indicated that these DEGs were mainly associated with lipid metabolism processes, including fatty acid β-oxidation and very-low-density lipoprotein particle remodeling (Figure 3E). For a comprehensive analysis, all expressed genes were subjected to GSEA. Similarly, GSEA plots of PPAR signaling pathway, fatty acid degradation, and fatty acid beta oxidation demonstrated the significant enrichment of related genes at the leading edge of the gene sets, indicating that these genes were predominantly upregulated in the CGA group (Figure 3F–H). Overall, the transcriptomic expression profiles confirm that CGA supplementation affects the lipid metabolism process in laying hens.

### 3.4. Effects of CGA on Hepatic Lipid Metabolism

**Figure 4:**
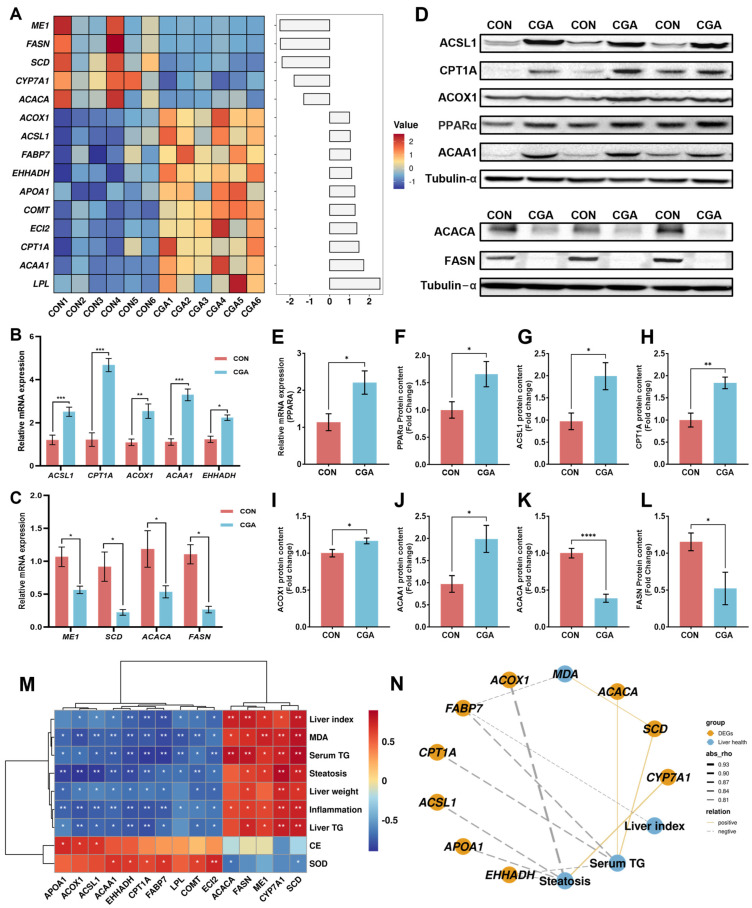
Effects of CGA on hepatic lipid metabolism. (**A**) Heatmap-bar plot. (**B**,**C**) The mRNA levels of DEGs in the livers of laying hens. (**E**) The mRNA levels of PPARA. (**D**,**F**–**L**) Western blot analysis of the differentially expressed proteins. (**M**) Correlation heatmap of DEGs and liver health indicators. (**N**) Correlation network of DEGs and liver health indicators. *, *p* < 0.05; **, *p* < 0.01; ***, *p* < 0.001, ****, *p* < 0.0001. The boxes in (**A**) represent the log2 fold change (log2FC) values. The original Western blot images for (**D**) are provided in the Supplementary Materials (Figures S1–S9).

Hepatic lipid metabolism-associated genes were screened out based on transcriptome sequencing to identify the key genes through which CGA regulates the process of hepatic lipid metabolism. As illustrated in Figure 4A, the expression of genes related to lipid metabolism, including *ME1*, *FASN*, *SCD*, *CYP7A1*, *ACACA*, *ACOX1*, *ACSL1*, *FABP7*, *EHHADH*, *APOA1*, *COMT*, *ECI2*, *CPT1A*, *ACAA1* and *LPL*, showed certain trends of change after dietary supplementation with CGA. Based on RNA-seq results, selected lipid metabolism-related genes were validated by qRT-PCR. CGA supplementation increased the mRNA expression of fatty acid oxidation-related genes, including *ACSL1*, *CPT1A*, *ACOX1*, *ACAA1*, and *EHHADH* (*p* < 0.05; Figure 4B). In contrast, genes associated with lipogenesis, including *ME1*, *SCD*, *ACACA*, and *FASN*, were downregulated in the CGA group (*p* < 0.05; Figure 4C). The expression trends were consistent with those observed in the RNA-Seq analysis, confirming the reliability of the transcriptome data. Western blot analysis showed similar changes at the protein level, including increased PPARα, ACSL1, CPT1A, ACOX1, and ACAA1 and decreased ACACA and FASN expression (*p* < 0.05, Figure 4D–L). These data showed that CGA supplementation was accompanied by the altered expression of hepatic lipid metabolism-related genes and proteins. We further quantified the mRNA and protein expression levels of PPARα, and the results were consistent with our expectations that CGA significantly increased its gene and protein expression (Figure 4D,E, *p* < 0.05).

To explore the potential mechanism by which dietary CGA regulates hepatic lipid metabolism in laying hens, Pearson correlation analysis was conducted between the liver lipid metabolism-related indicators and DEGs (Figure 4M). Briefly, the degree of hepatic steatosis and serum TG content were strongly negatively correlated with the expression of genes related to fatty acid oxidation and transport and were significantly positively correlated with genes related to fatty acid and bile acid synthesis (Figure 4N).

### 3.5. Serum Metabolomic Changes

**Figure 5:**
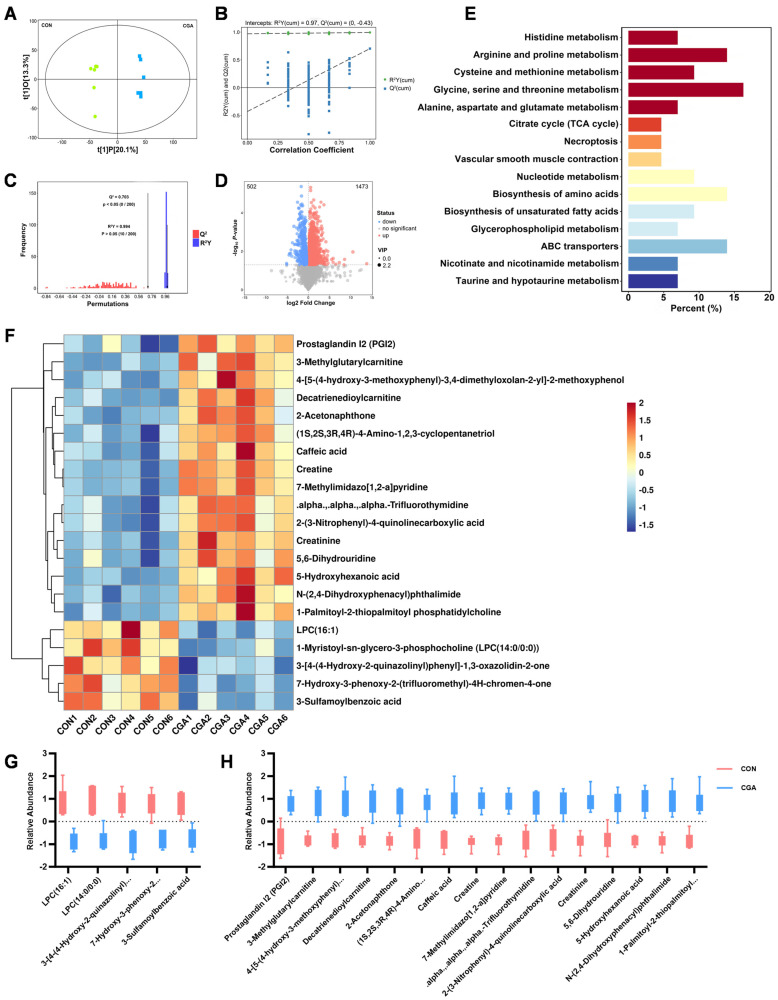
Effects of CGA on serum metabolomics. (**A**) OPLS-DA score scatter plot. Dot plot (**B**) and bar chart (**C**) of permutation test for OPLS-DA model. (**D**) Volcano plot of DAMs. (**E**) Top 15 KEGG enrichment pathways. (**F**) DAMs heatmap. The expression level change (Z-scored original value) of downregulated (**G**) and upregulated metabolites (**H**).

Serum untargeted LC-MS detected 9411 metabolic features across all samples. The OPLS-DA score plot distinctly showed a separation between the CON and CGA groups (Figure 5A), indicating that the CGA diet had a significant impact on the serum metabolites of laying hens. The OPLS-DA permutation test is used to verify the robustness and reliability of the OPLS-DA model. As shown in Figure 5B,C, the model has good fitting accuracy and prediction performance and can be used for subsequent analysis. In total, 1975 DAMs were identified among the serum metabolites between the CGA and CON groups, with 1473 upregulated and 502 downregulated DAMs in the CGA group (Figure 5D). KEGG analysis indicated that among the top 15 signaling pathways in which DAMs were mainly enriched, glycerophospholipid metabolism and biosynthesis of unsaturated fatty acids were related to fat metabolism. In addition, dietary CGA affects signaling pathways including amino acid metabolism, TCA cycle, nicotinic acid and nicotinamide metabolism, and taurine and hypo-taurine metabolism in the liver of laying hens (Figure 5E). Filtering (VIP > 1, FDR-adjusted *p*-value < 0.05) identified 21 differential metabolites, with 16 increased (e.g., caffeic acid, 5-Hydroxyhexanoic acid) and 5 decreased (e.g., LPC (16:1), LPC (14:0/0:0)) (Figure 5F–H).

**Figure 6:**
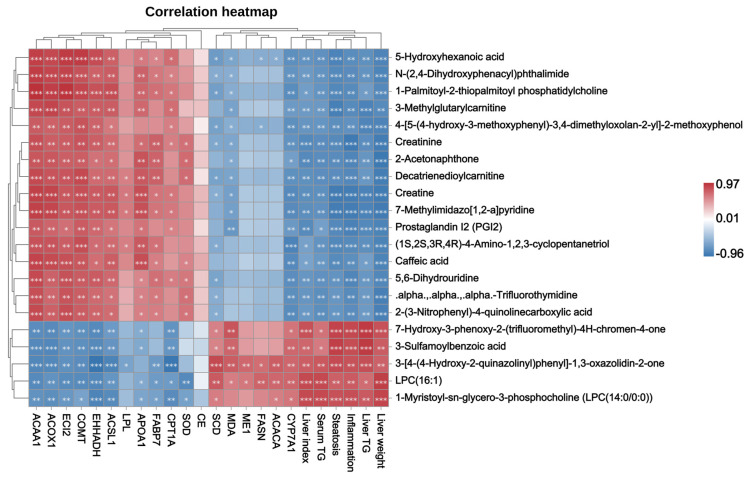
Correlation analysis of DAMs with hepatic lipid metabolism-related indicators and DEGs. *, *p* < 0.05; **, *p* < 0.01, ***, *p* < 0.001.

Similarly, we conducted a correlation analysis of 21 DAMs with hepatic lipid metabolism-related indicators and DEGs. As shown in Figure 6, many DAMs, such as caffeic acid, 3-Methylglutarylcarnitine, 1-Palmitoyl-2-thiopalmitoyl phosphatidylcholine, and 5-Hydroxyhexanoic acid, were positively correlated with indicators related to fatty acid oxidation and transport. DAMs like 3-Sulfamoylbenzoic acid, LPC (16:1), and LPC (14:0/0:0) were negatively correlated with fatty acid and bile acid production, as well as TG content.

### 3.6. Integrative Analysis

**Figure 7:**
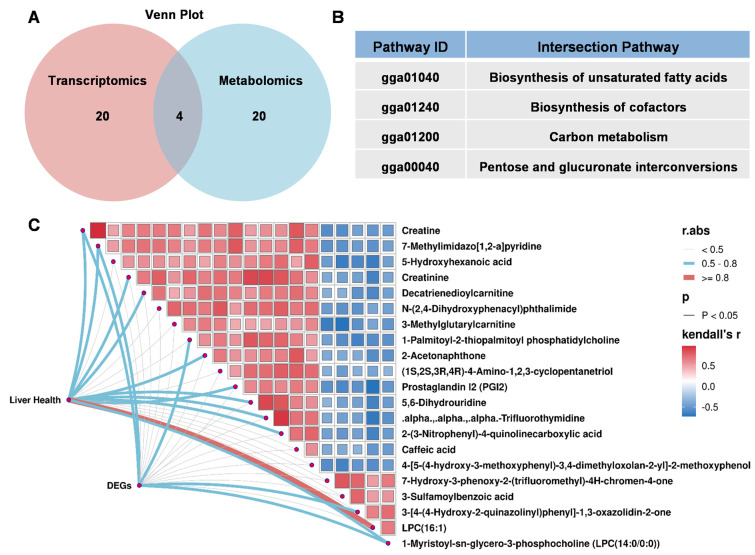
Integrative analysis. (**A**,**B**) Venn diagram illustrating the overlapping pathways between transcriptome pathways and metabolome pathways. (**C**) Correlation network heatmap of liver health-related indicators, DEGs, and DAMs.

Integrated pathway analysis of hepatic DEGs and serum metabolite annotations identified four overlapping pathways: biosynthesis of unsaturated fatty acids, biosynthesis of cofactors, carbon metabolism, and pentose and glucuronate interconversions (Figure 7A,B). The results of the correlation network heatmap showed that creatine, 7-Methylimidazo [1,2-a] pyridine, creatinine, decatrienedioyl-carnitine, 2-Acetonaphthone, PGI2, 5,6-Dihydrouridine, Trifluorothymidine, 2-(3-Nitrophenyl)-4-quinolinecarboxylic acid, LPC (16:1), and LPC (14:0/0:0) had a strong correlation with liver health indicators. Meanwhile, creatine, 7-Methylimidazo [1,2-a] pyridine, 1-Palmitoyl-2-thiopalmitoyl phosphatidyl-choline, 3-[4-(4-Hydroxy-2-quinazolinyl) phenyl]-1,3-oxazolidin-2-one, and LPC (14:0/0:0) were highly correlated with DEGs in the liver. Among them, creatine, 7-Methylimidazo [1,2-a] pyridine, and LPC (14:0/0:0) were strongly correlated with both DEGs and health indicators in the liver (Figure 7C). Because these correlations were based on a limited sample size, they should be interpreted as exploratory associations rather than evidence of causality.

## 4. Discussion

The present work addressed a previously underexplored question: whether dietary provision of chlorogenic acid can remodel hepatic lipid handling in commercially productive laying hens subjected to chronic thermal challenge during the physiologically demanding peak-production phase. The main findings were that 300 mg/kg CGA reduced hepatic lipid accumulation, improved selected antioxidant indices, and altered hepatic expression of genes and proteins involved in fatty acid oxidation and lipogenesis. Integrated transcriptomic and serum metabolomic analyses further suggested that CGA supplementation was associated with the coordinated regulation of PPARα-related lipid metabolism pathways and circulating lipid-related metabolites.

Dietary supplementation with CGA under heat stress conditions did not adversely affect the laying performance parameters, including the laying rate, average egg weight, or feed-egg ratio, throughout the 8-week trial (*p* > 0.05; Table 3). This is consistent with findings in aged breeder hens, where CGA (250 mg/kg) exerted no significant impact on egg production or feed efficiency [35]. In contrast, higher doses (600–800 mg/kg) in oxidative-stress models have occasionally improved the laying rate [45], suggesting that the moderate dose and stress intensity in the present study primarily stabilize rather than enhance productivity [32,45]. Regarding egg quality, CGA supplementation significantly improved the egg white height and resulted in higher Haugh unit values at the 4th week (*p* < 0.001; Table 4). These changes are attributable to CGA’s potent antioxidant properties, which likely protect ovomucin and other albumen proteins from heat-stress-induced oxidative degradation during formation [32,35]. At week 8, the yolk color score was elevated (*p* < 0.05), consistent with polyphenols enhancing carotenoid absorption, transport, or stability in the yolk [45]. A darker yolk color often correlates with perceived higher egg quality and consumer preference. Notably, the eggshell weight was transiently lower at week 4 (*p* = 0.022), yet other shell parameters remained comparable, and the difference disappeared by week 8. Heat stress impairs calcium homeostasis by suppressing carbonic anhydrase activity in the shell gland and downregulating intestinal calcium transport proteins (CaBP-D28k and Ca^2+^-ATPase) [15,46,47,48]. The metabolic reprogramming induced by CGA in the early supplementation phase may have transiently competed with calcium deposition in the shell gland, while the subsequent restoration of oxidative status and intestinal barrier function by week 8 likely recovered the calcium transport capacity, consistent with the disappearance of this effect [49,50,51]. From an industry perspective, given that eggshell thickness, strength, and breakage rate were unaffected, this transient reduction is unlikely to result in significant economic losses under commercial conditions. Nevertheless, during the early phase of nutritional intervention, eggshell quality parameters should be closely monitored in peak-laying flocks under heat stress, and adequate dietary calcium and vitamin D_3_ supplementation is recommended as a precautionary measure. Importantly, no persistent negative effects of CGA on eggshell quality have been reported in the literature [35,45], consistent with our week 8 findings. Future studies incorporating serial measurements of serum calcium and CaBP-D28k expression will be needed to further elucidate the mechanism underlying this transient effect. Overall, these results indicate that CGA supplementation in heat-stressed peak-laying hens maintains production stability while selectively enhancing internal egg quality attributes, supporting its potential as a safe nutritional strategy for mitigating environmental stress without compromising output.

CGA supplementation significantly ameliorated hepatic steatosis, reducing macroscopic yellowish discoloration, liver weight, liver index, and the hepatic and serum TG levels (*p* < 0.05; Figure 2). Interestingly, CGA increased the hepatic CE content without significantly altering the TC or FC levels. The concurrent reduction in TG and elevation in CE suggests a selective remodeling of hepatic lipid composition rather than a generalized suppression of lipid accumulation. Mechanistically, CGA activates the AMPK–PPARα signaling axis to promote fatty acid β-oxidation and suppress de novo lipogenesis, thereby preferentially reducing hepatic TG synthesis and deposition [32,45,52]. As the TG burden is relieved, the relative availability of free cholesterol and acyl-CoA substrates may increase, potentially driving enhanced acyl-CoA:cholesterol acyltransferase (ACAT)-mediated cholesterol esterification [53]. Given that excess free cholesterol is inherently cytotoxic—capable of disrupting membrane fluidity, inducing oxidative damage, and promoting endoplasmic reticulum stress [54,55,56]—its conversion to CE and storage in lipid droplets more likely represents an adaptive hepatoprotective response rather than pathological cholesterol dysregulation. Nevertheless, hepatic cholesterol homeostasis is coordinately regulated by synthesis, esterification, bile acid conversion, and lipoprotein assembly [56,57]; as ACAT activity, markers of bile acid metabolism, and VLDL export were not directly measured, mechanistic conclusions remain to be confirmed by future studies. Histological evidence (H&E and Oil Red O) confirmed fewer and smaller lipid droplets, with lower steatosis and inflammation scores. Concurrently, hepatic SOD activity increased, while MDA decreased (*p* < 0.05), indicating enhanced antioxidant capacity without altering GSH, GSH-PX, or CAT. These hepatoprotective effects break the vicious cycle whereby peak-laying metabolic demand and heat stress generate ROS that exacerbate lipid accumulation, mirroring CGA’s efficacy in H_2_O_2_-challenged laying hens and in mammalian MAFLD models, where CGA reduces oxidative stress and steatosis [45,58,59]. However, it should be acknowledged that avian hepatic lipid metabolism differs from that of mammals in several key aspects: in laying hens, the liver is the predominant site of de novo lipogenesis, and avian hepatocytes exhibit distinct lipoprotein assembly and VLDL secretion characteristics compared with mammalian hepatocytes. Moreover, the regulatory sensitivity of PPARα and AMPK signaling to dietary polyphenols may vary across species. Therefore, mechanistic inferences drawn from mammalian models should be applied to the avian context with caution, and species-specific validation remains necessary.

The transcriptomic and validation results indicated that CGA supplementation was associated with the increased expression of PPARα and several canonical fatty acid oxidation-related genes, including *ACSL1*, *CPT1A*, *ACOX1*, *ACAA1*, and *EHHADH*. These genes participate in fatty acid activation, mitochondrial fatty acid transport, peroxisomal β-oxidation, and downstream acetyl-CoA generation. In parallel, CGA decreased the expression of lipogenic markers such as *ACACA*, *FASN*, *SCD*, and *ME1*, suggesting a metabolic shift from lipid synthesis toward fatty acid utilization. While our gene expression and protein level data consistently showed PPARα upregulation alongside its downstream targets, we acknowledge that this study provides correlative rather than causal evidence for PPARα mediation of CGA’s effects. Direct mechanistic validation through PPARα antagonist studies (e.g., GW6471) or hepatocyte-specific PPARα knockout models would be required to establish causality [60,61]. Additionally, chromatin immunoprecipitation sequencing (ChIP-seq) could confirm whether CGA treatment enhances PPARα binding to target gene promoters [62]. These functional studies represent important future directions to definitively establish the PPARα-dependent mechanisms of CGA action. Collectively, the observed upregulation of β-oxidation genes and downregulation of lipogenic markers are consistent with a coordinated metabolic shift that may underlie the amelioration of FLHS observed in the present study, supporting the potential of CGA as a dietary strategy for the prevention and mitigation of FLHS in peak-laying hens under heat stress.

Serum metabolomic analysis revealed clear group separation in the OPLS-DA score plot, identifying 1975 differentially abundant metabolites (DAMs), of which 1473 were upregulated and 502 downregulated in the CGA group (Figure 5). Among the 21 key filtered DAMs (VIP > 1, FDR-adjusted *p* < 0.05), those with established or putative biological relevance to hepatic lipid metabolism, fatty acid oxidation, or phospholipid remodeling were prioritized for in-depth biological interpretation. Upregulation of caffeic acid, the primary bioactive metabolite of CGA, is consistent with effective intestinal hydrolysis and systemic bioavailability and aligns with its documented hepatoprotective and lipid-modulating activities [63]; however, the relative contributions of gut microbiota biotransformation and direct intestinal absorption to circulating caffeic acid levels cannot be distinguished in the present study. Increases in metabolites such as 5-Hydroxyhexanoic acid further support enhanced fatty acid catabolism and energy metabolism [64]. Several LPC species, including LPC (16:1) and LPC (14:0/0:0), were decreased in the serum of CGA-supplemented hens. LPCs are bioactive lipid molecules with species-specific functions, and their biological effects depend on acyl-chain composition, tissue distribution, and transport pathways. Recent evidence indicates that blood-derived PUFA-containing LPCs can contribute to hepatic phospholipid pools and hepatoprotection under overnutrition, whereas other LPC species may be associated with inflammatory or lipotoxic signaling [65,66]. Therefore, the observed reduction in selected serum LPC species should not be interpreted as uniformly beneficial. Because hepatic LPC, PC, PE, and Mfsd2a-mediated LPC transport were not measured in this study, the role of LPC remodeling in CGA-mediated hepatic protection remains speculative and warrants targeted lipidomic validation. KEGG enrichment in glycerophospholipid metabolism, unsaturated fatty acid biosynthesis, TCA cycle, and amino acid metabolism pathways is consistent with the transcriptomic activation of β-oxidation, suggesting a systemic metabolic signature associated with CGA’s protective action on hepatic lipid metabolism. Nevertheless, it should be noted that serum metabolites reflect systemic metabolic changes arising from multiple sources, including intestinal absorption, gut microbiota metabolism, hepatic output, and peripheral tissue mobilization; as these associations are correlative rather than causal, and as no gut microbiota or intestinal permeability analyses were performed, mechanistic conclusions regarding gut–liver axis involvement remain to be confirmed by future integrated multi-omics studies incorporating intestinal and microbial profiling.

Integrated correlation analyses (Figure 6) linked hepatic DEGs (e.g., PPARα, CPT1A) and health indicators strongly to serum metabolites (e.g., creatine, specific phospholipids, caffeic acid), confirming that transcriptional reprogramming drives systemic metabolic changes. The serum profile thus serves as a reliable readout of CGA’s hepatic actions via the gut–liver axis. Integrative analysis of the hepatic transcriptome and serum metabolome identified four overlapping pathways biosynthesis of unsaturated fatty acids, biosynthesis of cofactors, carbon metabolism, and pentose and glucuronate interconversions, demonstrating the coordinated multi-level regulation of lipid handling and energy metabolism by CGA (Figure 7). Correlation network heatmaps revealed robust interconnections among liver health indicators, DEGs, and key DAMs, with creatine, creatinine, caffeic acid, and specific carnitine phospholipid derivatives positively associated with fatty acid oxidation genes and antioxidant capacity, while LPC (14:0/0:0) and related species showed strong negative correlations with lipogenic indicators and TG levels. These integrated findings illustrate how CGA-induced transcriptional reprogramming (primarily via PPARα) is faithfully mirrored in the serum metabolome, confirming a mechanistic link between hepatic gene expression and systemic metabolic outcomes through the gut–liver axis. This multi-omics convergence aligns with recent integrative studies showing CGA attenuates oxidative stress and lipid disorders in laying hens via microbiome–metabolite–transcriptome crosstalk along the gut–liver axis [51] and in mammalian obesity models where serum metabolite–liver axis modulation underlies CGA’s lipid-ameliorating effects [67].

From a veterinary and production perspective, improving hepatic lipid metabolism during the peak laying period may help maintain liver health and productive longevity under high-temperature conditions. Compared with late-stage intervention, nutritional prevention during the metabolically active peak-laying phase may be more beneficial for reducing hepatic lipid overload. This study has several limitations. First, the stability of CGA in complete feed under the storage and experimental heat-stress conditions was not directly verified by chemical analysis. Given that CGA is susceptible to oxidative degradation, future studies should incorporate HPLC-based quantification of the feed CGA content at multiple time points to confirm that the intended supplementation level was maintained throughout the experimental period. Second, only hens maintained under heat-stress conditions were included; therefore, the study cannot distinguish CGA-specific effects under heat stress from its general lipid-modulating effects under thermoneutral conditions. A future 2 × 2 factorial design including thermoneutral and heat-stress environments with or without CGA is needed. Third, only one CGA dose was tested, and the 8-week duration does not allow the assessment of long-term productivity, eggshell quality, or safety; systematic dose–response studies and long-term trials covering the full laying cycle are needed before practical feeding recommendations can be established. Fourth, thermoregulatory biomarkers (e.g., rectal temperature, corticosterone, HSP70) were not measured beyond environmental THI indices, and direct measurements of thermophysiological responses—including individual heat stress intensity quantification—were therefore not obtained; mitochondrial function and fatty acid oxidation flux were likewise not directly assessed through enzymatic activity assays or stable isotope-based metabolic flux analyses, and lipid flux was not quantified. Fifth, although PPARα-related genes and proteins were upregulated, direct PPARα transcriptional activity was not assessed through reporter assays or chromatin immunoprecipitation, and causality between CGA supplementation and enhanced fatty acid oxidation was not experimentally established; accordingly, mechanistic interpretations regarding PPARα activation and β-oxidation should be regarded as hypotheses requiring direct experimental validation in future work. Sixth, hepatic targeted lipidomics, gut microbiota, and intestinal barrier measurements were not performed, limiting the mechanistic interpretation of LPC remodeling and gut–liver interactions. Additionally, the sample size for omics analyses was relatively limited, and metabolic evaluations were restricted to two time points (weeks 4 and 8), constraining the statistical power of omics findings and the characterization of the full temporal trajectory of CGA-induced metabolic adaptations.

## 5. Conclusions

In conclusion, dietary supplementation with 300 mg/kg CGA was associated with reduced hepatic lipid accumulation and improved selected antioxidant indices in peak-laying hens maintained under heat-stress conditions. These effects were accompanied by upregulation of PPARα-associated fatty acid oxidation genes and proteins, downregulation of lipogenic markers, and remodeling of serum metabolic features. Although direct mechanistic validation remains to be established, CGA may represent a promising nutritional strategy for improving hepatic metabolic health in laying hens exposed to high-temperature production environments.

## Figures and Tables

**Table 1:** Composition and nutrient levels of the experimental basal diet.

Composition (Air-Dry Basis) %	Basal Diet
Corn	62
Soybean meal	24
Soybean oil	0.5
Limestone	9
Premix ^1^	4.5
Total	100
**Calculated Nutrient Levels**	
Metabolizable energy, kcal/kg	2610
Crude protein	16.02%
Calcium	3.87%
Available phosphorus	0.31%
Lysine	0.74%
Methionine	0.38%

^1^ The premix consists of 1% stone powder, 1.2% calcium hydrogen phosphate, 0.12% DL-methionine, 1% composite premix, 0.3% salt (feed grade), and 0.88% carrier.

**Table 2:** Primers for RT-qPCR analysis.

Gene	Primer Sequence (5′–3′) ^1^	Length (bp)	GenBank Number
*ACSL1*	F: AGGCTGTCCTGGTGGTGATT R: CAGCAGCAGCAGCAGTAGTG	198	NM_001039615.2
*CPT1A*	F: TCGTCTGCCATGACTGGTG R: GCTGTGGTGTCTGACTCGTT	126	XM_040700878.2
*ACOX1*	F: ATGTCACGTTCACCCCATCC R: AGGTAGGAGACCATGCCAGT	145	NM_001006205.2
*ACAA1*	F: GCCAAGCTGCTGTTTGAGAA R: AGGATGCGGCTGATGTTTTG	165	NM_001006192.2
*EHHADH*	F: TGCTGGTGGCCTCTATCTGG R: CCTGCTTCTCGATGGCCTCT	201	NM_001006189.2
*ME1*	F: GCTGCCTCTGCTCCTCTCCT R: GCCTCCTCCTCCTCCTCCTC	187	NM_001006523.2
*SCD*	F: AGCAGAACGAGGCATGGTAG R: GGATCAGCGTCAGCCCAATA	80	NM_204890.2
*ACACA*	F: CCGAGAACCCAAAACTACCAG R: GCCAGCAGTCTGAGCCACTA	124	NM_205505.1
*FASN*	F: TGGCATACGAACTGGCTACC R: CCAACTGGTCCGAGCTTCAA	128	NM_205155.4
*PPARA*	F: GTGTGGCTGCTGCTTAGAGA R: CCGCATTTTGAAGGACGGTT	155	NM_001001464.1
*β-Actin*	F: TGCGTGACATCAAGGAGAAG R: TGCCAGGGTACATTGTGGTA	142	NM_205518

^1^ F, forward; R, reverse.

**Table 3:** The effects of dietary CGA inclusion on laying performance.

Items	Groups		*p*-Value
	CON	CGA	
1–4 weeks			
Laying rate, %	80.34 ± 1.09	79.32 ± 2.23	0.564
Average egg weight, g	54.89 ± 0.29	54.99 ± 0.29	0.257
Feed-egg ratio, g/g	2.93 ± 0.05	2.97 ± 0.09	0.289
5–8 weeks			
Laying rate, %	78.78 ± 1.19	77.52 ± 1.37	0.851
Average egg weight, g	56.45 ± 0.38	56.52 ± 0.29	0.563
Feed-egg ratio, g/g	2.94 ± 0.05	2.93 ± 0.03	0.886
1–8 weeks			
Laying rate, %	79.56 ± 0.86	78.42 ± 1.56	0.675
Average egg weight, g	55.67 ± 0.32	55.75 ± 0.28	0.449
Feed-egg ratio, g/g	2.93 ± 0.04	2.98 ± 0.06	0.373

Note: Data are represented as mean ± SEM; *n* = 6.

**Table 4:** The effects of dietary CGA inclusion on egg quality.

Items	Groups		*p*-Value
	CON	CGA	
4 weeks			
Egg weight, g	57.71 ± 0.76	55.90 ± 0.79	0.107
Albumen height, mm	7.72 ± 0.21b	9.52 ± 0.35a	<0.001
Haugh unit	89.07 ± 0.82b	100.01 ± 0.83a	<0.001
Yolk color	12.72 ± 0.13	12.67 ± 0.11	0.790
Yolk weight, g	14.05 ± 0.15	14.01 ± 0.13	0.809
Yolk index	24.71 ± 0.32	24.97 ± 0.42	0.626
Eggshell weight, g	7.50 ± 0.08a	7.17 ± 0.11b	0.022
Eggshell index	13.00 ± 0.14	12.85 ± 0.16	0.471
Albumen weight, g	35.28 ± 0.53	35.01 ± 0.64	0.745
Albumen index	61.91 ± 0.32	62.27 ± 0.29	0.395
Eggshell thickness, mm	0.37 ± 0.01	0.37 ± 0.00	0.304
Eggshell strength, kg/cm^2^	4.11 ± 0.01b	4.13 ± 0.02a	0.106
8 weeks			
Egg weight, g	56.67 ± 0.72	56.61 ± 0.82	0.962
Albumen height, mm	10.25 ± 0.17	10.11 ± 0.19	0.579
Haugh unit	100.84 ± 0.82	100.82 ± 0.78	0.988
Yolk color	12.89 ± 0.10b	13.30 ± 0.11a	0.009
Yolk weight, g	14.85 ± 0.17	14.56 ± 0.21	0.305
Yolk index	25.87 ± 0.39	26.29 ± 0.43	0.473
Eggshell weight, g	7.59 ± 0.11	7.44 ± 0.11	0.386
Eggshell index	13.35 ± 0.12	13.21 ± 0.18	0.521
Albumen weight, g	34.67 ± 0.67	33.66 ± 0.72	0.313
Albumen index	60.54 ± 0.40	60.49 ± 0.50	0.939
Eggshell thickness, μm	0.37 ± 0.01	0.37 ± 0.00	0.238
Eggshell strength, kg/cm^2^	4.20 ± 0.04	4.13 ± 0.03	0.153

Note: Data are represented as mean ± SEM; different letters indicate differences between treatments (*p* < 0.05); *n* = 30.

## Data Availability

The data presented in this study are available on request from the corresponding author. The data are not publicly available due to ethical restrictions.

## Supplementary Materials

The following supporting information can be downloaded at https://www.mdpi.com/article/10.3390/biology15120917/s1.
