# Supplementary material for: Dietary Chlorogenic Acid Attenuates Hepatic Lipid Accumulation and Reprograms Lipid Metabolism in Heat-Stressed Laying Hens: Integrated Transcriptomic and Metabolomic Analyses
Source: Biology (Basel). 2026 Jun 12;15(12):917. doi: 10.3390/biology15120917 (PMC13295461; doi:10.3390/biology15120917)
Supplement: Supplementary file 1 [file biology-15-00917-s001.zip › biology-4361279-supplementary.pdf]

Supplementary Materials

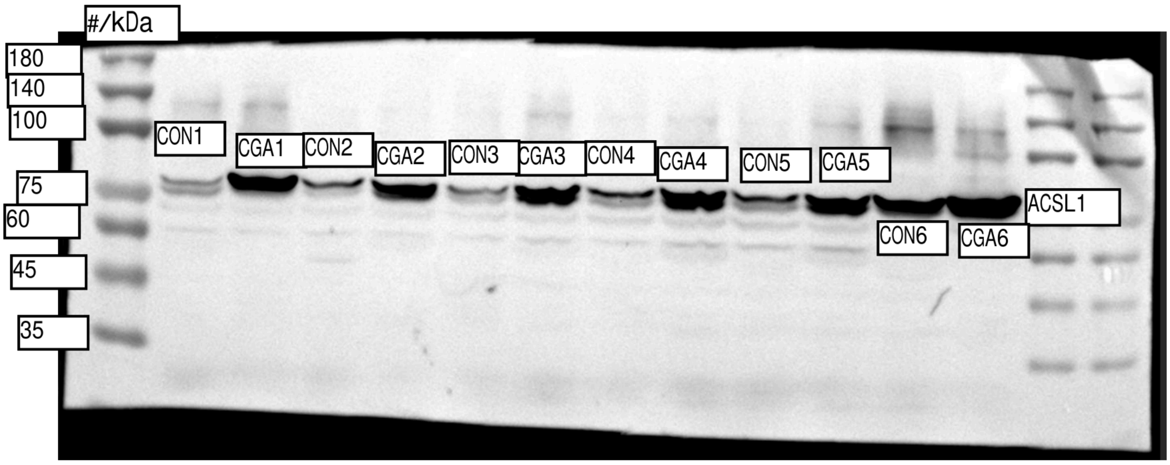

Figure S1. Western blot membrane of ACSL1 (~70 kDa) protein detected with anti-ACSL1 (13989-1-AP; 1:1000; Proteintech, Rosemont, IL, USA) antibody. Protein separation was achieved by denaturing polyacrylamide gel electro-phoresis, after which proteins were electrotransferred onto nitrocellulose membranes under constant current conditions. After incubating with the horseradish peroxidase (HRP)-labeled goat anti-rabbit IgG (1:5000), the proteins were determined using the ultra-sensitive ECL chemiluminescence kit (RM00020P, ABclonal, CN) by the digital imaging equipment (GE Healthcare, Chicago, IL, USA). #Weight marker (molecular weight in kDa): ABclonal ColorMixed Protein Marker 180 (10–180 kDa), catalog number: RM19001; and ABclonal ColorMixed Protein Marker 310 (10–310 kDa), catalog number: RM02943. Blot images, prior to the densitometry readings, were converted to grayscale with ImageJ (ImageJ v.1.49, National Institutes of Health, Maryland, USA) as follows: Image -> Type –> 8 bit, next: Image -> Adjust–> Brightness/Contrast –> Auto.

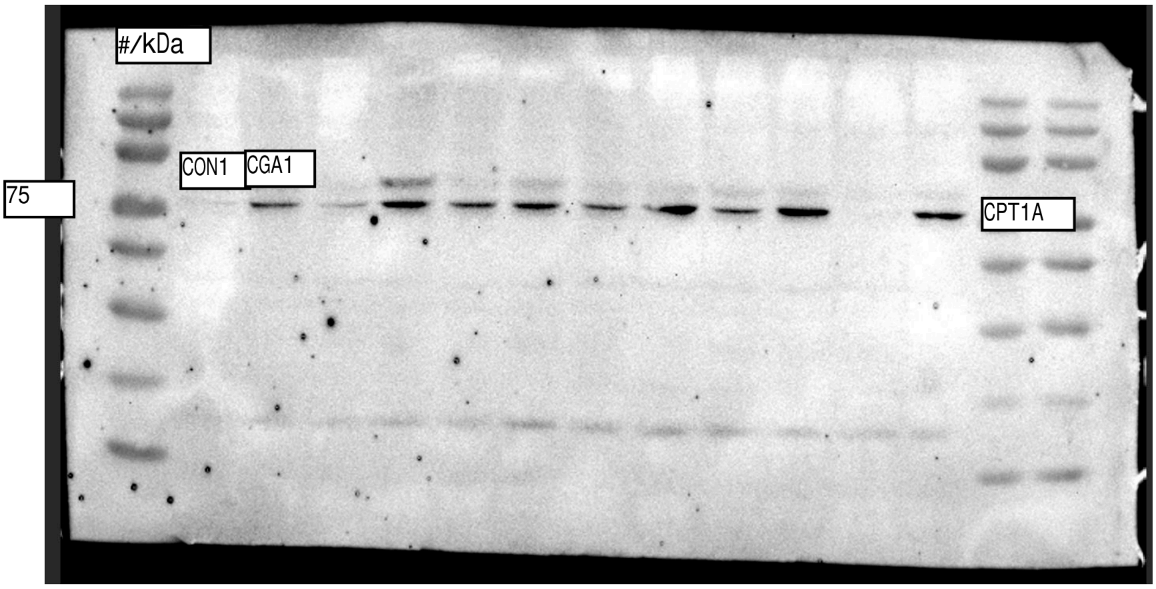

Figure S2. Western blot membrane of CPT1A (~80 kDa) protein detected with anti-CPT1A (15184-1-AP; 1:1000; Proteintech, Rosemont, IL, USA) antibody. Protein separation was achieved by denaturing polyacrylamide gel electro-phoresis, after which proteins were electrotransferred onto nitrocellulose membranes under constant current conditions. After incubating with the horseradish peroxidase (HRP)-labeled goat anti-rabbit IgG (1:5000), the proteins were determined using the ultra-sensitive ECL chemiluminescence kit (RM00020P, ABclonal, CN) by the digital imaging equipment (GE Healthcare, Chicago, IL, USA). #Weight marker (molecular weight in kDa): ABclonal ColorMixed Protein Marker 180 (10–180 kDa), catalog number: RM19001; and ABclonal ColorMixed Protein Marker 310 (10–310 kDa), catalog number: RM02943. Blot images, prior to the densitometry readings, were converted to grayscale with ImageJ (ImageJ v.1.49, National Institutes of Health, Maryland, USA) as follows: Image -> Type –> 8 bit, next: Image -> Adjust–> Brightness/Contrast –> Auto.

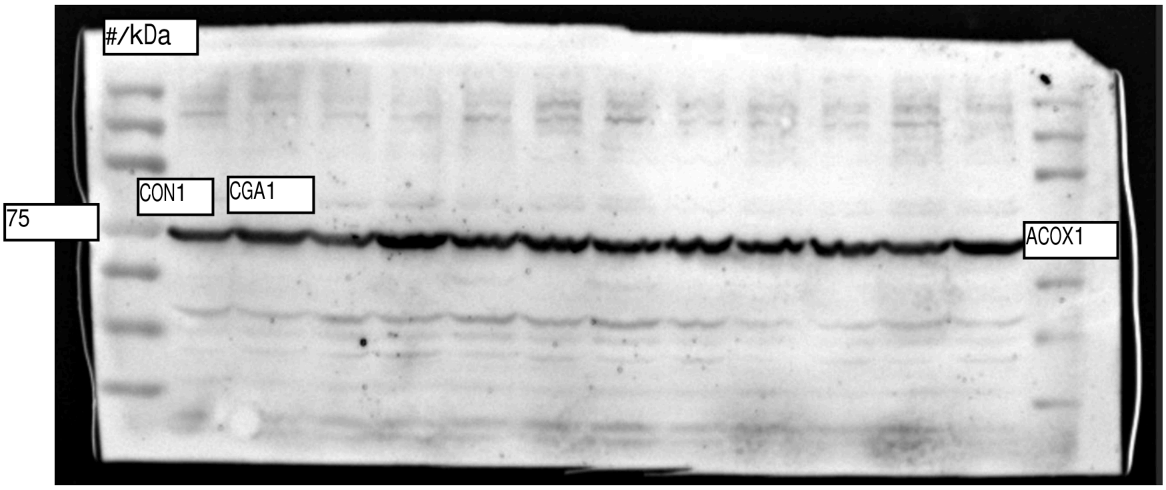

Figure S3. Western blot membrane of ACOX1 (~75 kDa) protein detected with anti-ACOX1 (A8091; 1:1000; ABclonal, Wuhan, China) antibody. Protein separation was achieved by denaturing polyacrylamide gel electro-phoresis, after which proteins were electrotransferred onto nitrocellulose membranes under constant current conditions. After incubating with the horseradish peroxidase (HRP)-labeled goat anti-rabbit IgG (1:5000), the proteins were determined using the ultra-sensitive ECL chemiluminescence kit (RM00020P, ABclonal, CN) by the digital imaging equipment (GE Healthcare, Chicago, IL, USA). #Weight marker (molecular weight in kDa): ABclonal ColorMixed Protein Marker 180 (10–180 kDa), catalog number: RM19001; and ABclonal ColorMixed Protein Marker 310 (10–310 kDa), catalog number: RM02943. Blot images, prior to the densitometry readings, were converted to grayscale with ImageJ (ImageJ v.1.49, National Institutes of Health, Maryland, USA) as follows: Image -> Type –> 8 bit, next: Image -> Adjust–> Brightness/Contrast –> Auto.

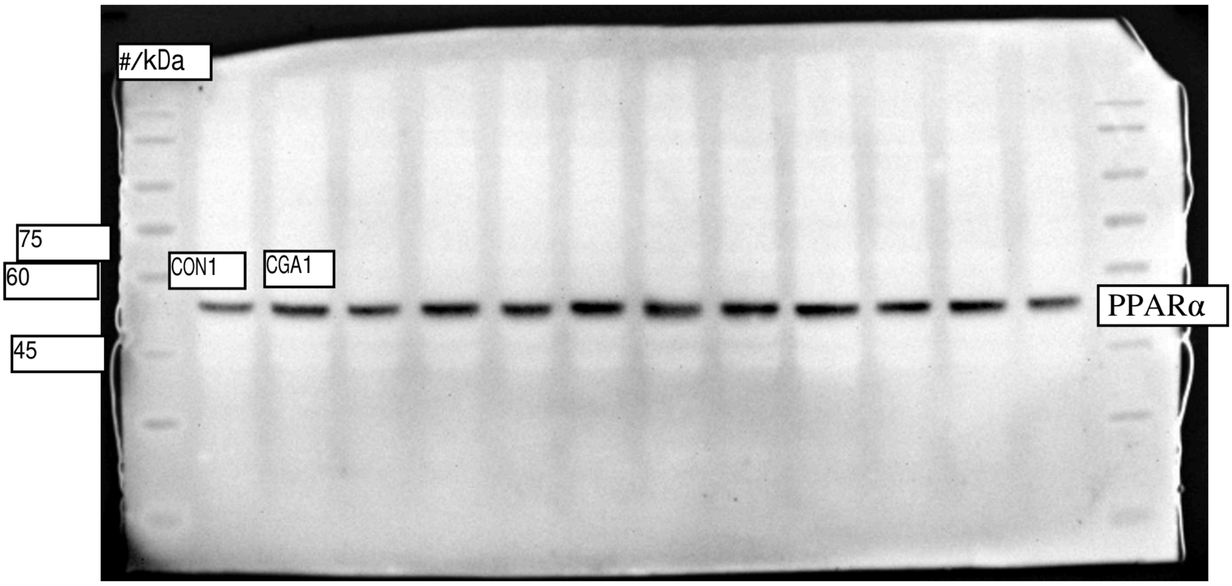

Figure S4. Western blot membrane of PPARα (~52 kDa) protein detected with anti-PPARα (A25296; 1; 1:1000; ABclonal, Wuhan, China) antibody. Protein separation was achieved by denaturing polyacrylamide gel electro-phoresis, after which proteins were electrotransferred onto nitrocellulose membranes under constant current conditions. After incubating with the horseradish peroxidase (HRP)-labeled goat anti-rabbit IgG (1:5000), the proteins were determined using the ultra-sensitive ECL chemiluminescence kit (RM00020P, ABclonal, CN) by the digital imaging equipment (GE Healthcare, Chicago, IL, USA). #Weight marker (molecular weight in kDa): ABclonal ColorMixed Protein Marker 180 (10–180 kDa), catalog number: RM19001; and ABclonal ColorMixed Protein Marker 310 (10–310 kDa), catalog number: RM02943. Blot images, prior to the densitometry readings, were converted to grayscale with ImageJ (ImageJ v.1.49, National Institutes of Health, Maryland, USA) as follows: Image -> Type –> 8 bit, next: Image -> Adjust–> Brightness/Contrast –> Auto.

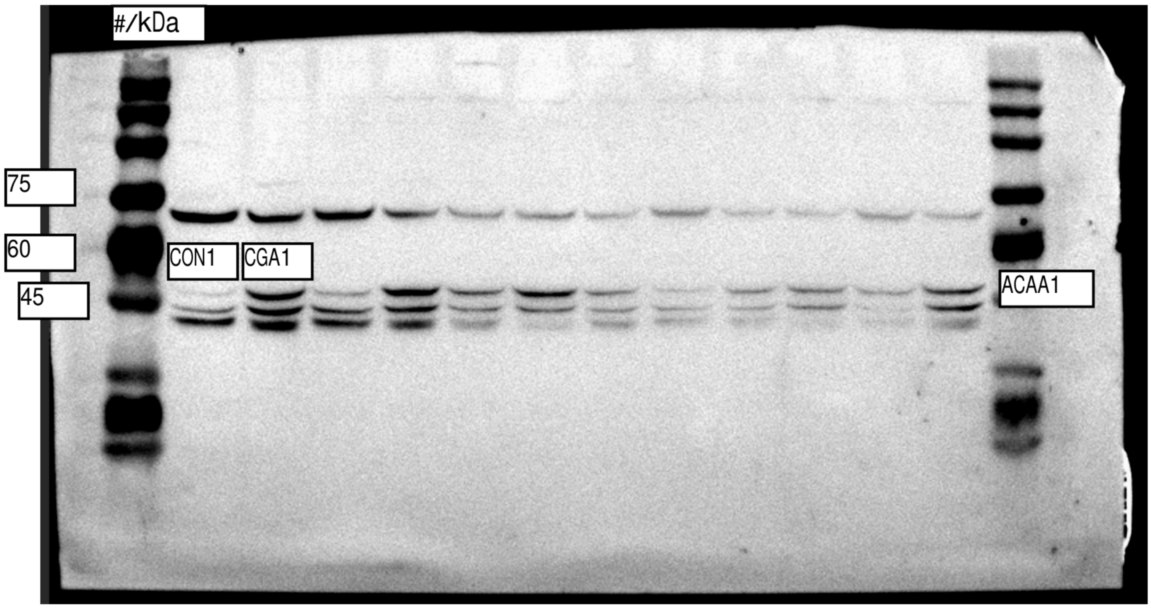

Figure S5. Western blot membrane of ACAA1 (~44 kDa) protein detected with anti-ACAA1 (A7422; 1; 1:1000; ABclonal, Wuhan, China) antibody. Protein separation was achieved by denaturing polyacrylamide gel electro-phoresis, after which proteins were electrotransferred onto nitrocellulose membranes under constant current conditions. After incubating with the horseradish peroxidase (HRP)-labeled goat anti-rabbit IgG (1:5000), the proteins were determined using the ultra-sensitive ECL chemiluminescence kit (RM00020P, ABclonal, CN) by the digital imaging equipment (GE Healthcare, Chicago, IL, USA). #Weight marker (molecular weight in kDa): ABclonal ColorMixed Protein Marker 180 (10–180 kDa), catalog number: RM19001; and ABclonal ColorMixed Protein Marker 310 (10–310 kDa), catalog number: RM02943. Blot images, prior to the densitometry readings, were converted to grayscale with ImageJ (ImageJ v.1.49, National Institutes of Health, Maryland, USA) as follows: Image -> Type –> 8 bit, next: Image -> Adjust–> Brightness/Contrast –> Auto.

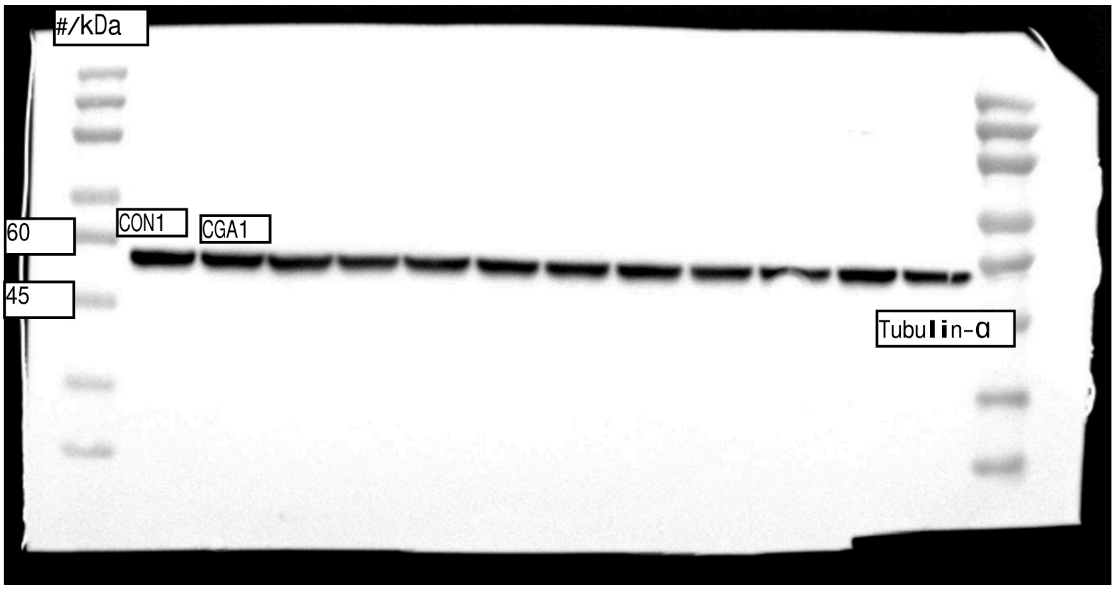

Figure S6. Western blot membrane of Tubulin-α (~55 kDa) protein detected with anti-Tubulin-α (BS1699; 1; 1:10000; Bioworld, USA) antibody. Protein separation was achieved by denaturing polyacrylamide gel electro-phoresis, after which proteins were electrotransferred onto nitrocellulose membranes under constant current conditions. After incubating with the horseradish peroxidase (HRP)-labeled goat anti-rabbit IgG (1:5000), the proteins were determined using the ultra-sensitive ECL chemiluminescence kit (RM00020P, ABclonal, CN) by the digital imaging equipment (GE Healthcare, Chicago, IL, USA). #Weight marker (molecular weight in kDa): ABclonal ColorMixed Protein Marker 180 (10–180 kDa), catalog number: RM19001; and ABclonal ColorMixed Protein Marker 310 (10–310 kDa), catalog number: RM02943. Blot images, prior to the densitometry readings, were converted to grayscale with ImageJ (ImageJ v.1.49, National Institutes of Health, Maryland, USA) as follows: Image -> Type –> 8 bit, next: Image -> Adjust–> Brightness/Contrast –> Auto.

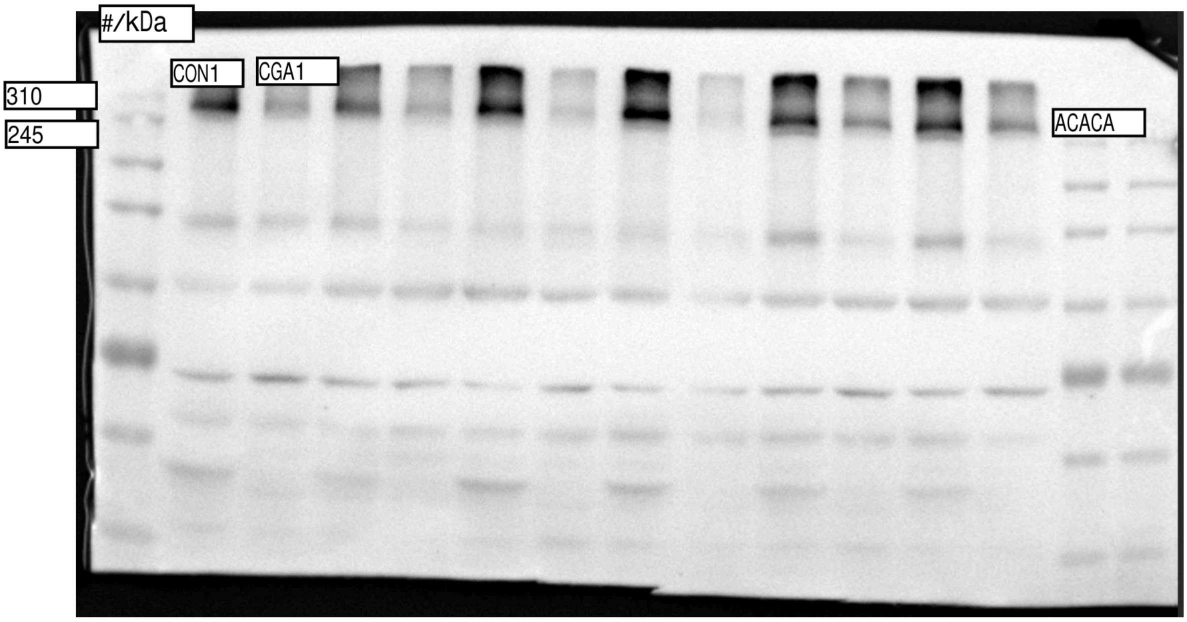

Figure S7. Western blot membrane of ACACA (~250 kDa) protein detected with anti-ACACA (A15606; 1; 1:1000; ABclonal, Wuhan, China) antibody. Protein separation was achieved by denaturing polyacrylamide gel electro-phoresis, after which proteins were electrotransferred onto nitrocellulose membranes under constant current conditions. After incubating with the horseradish peroxidase (HRP)-labeled goat anti-rabbit IgG (1:5000), the proteins were determined using the ultra-sensitive ECL chemiluminescence kit (RM00020P, ABclonal, CN) by the digital imaging equipment (GE Healthcare, Chicago, IL, USA). #Weight marker (molecular weight in kDa): ABclonal ColorMixed Protein Marker 180 (10–180 kDa), catalog number: RM19001; and ABclonal ColorMixed Protein Marker 310 (10–310 kDa), catalog number: RM02943. Blot images, prior to the densitometry readings, were converted to grayscale with ImageJ (ImageJ v.1.49, National Institutes of Health, Maryland, USA) as follows: Image -> Type –> 8 bit, next: Image -> Adjust–> Brightness/Contrast –> Auto.

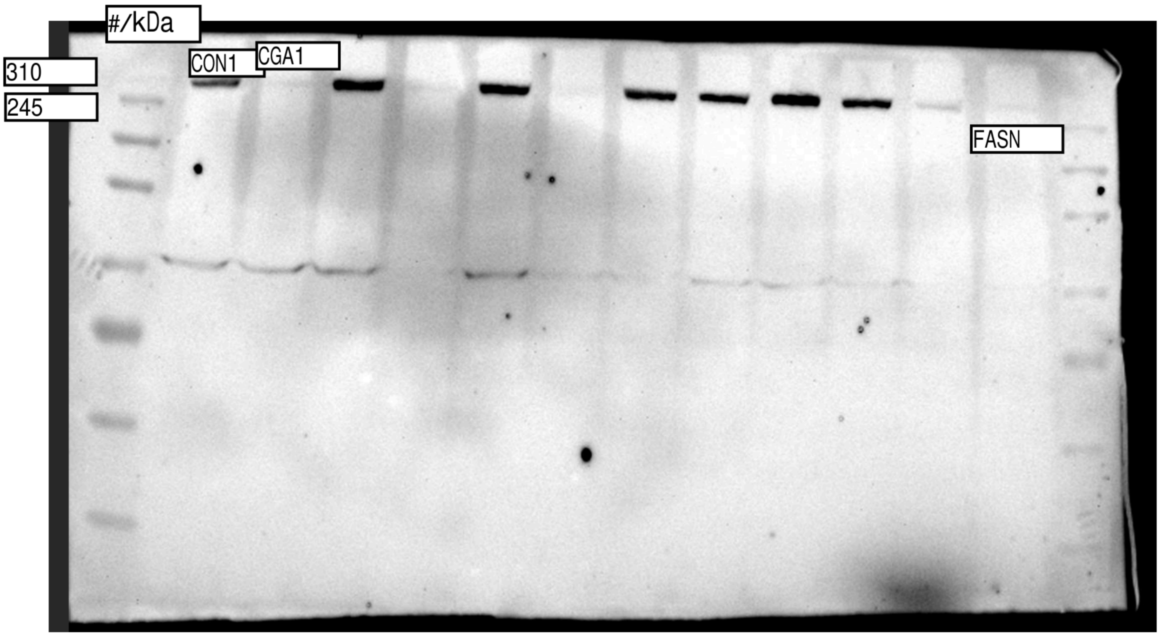

Figure S8. Western blot membrane of FASN (~273 kDa) protein detected with anti-FASN (A0461; 1; 1:1000; ABclonal, Wuhan, China) antibody. Protein separation was achieved by denaturing polyacrylamide gel electro-phoresis, after which proteins were electrotransferred onto nitrocellulose membranes under constant current conditions. After incubating with the horseradish peroxidase (HRP)-labeled goat anti-rabbit IgG (1:5000), the proteins were determined using the ultra-sensitive ECL chemiluminescence kit (RM00020P, ABclonal, CN) by the digital imaging equipment (GE Healthcare, Chicago, IL, USA). #Weight marker (molecular weight in kDa): ABclonal ColorMixed Protein Marker 180 (10–180 kDa), catalog number: RM19001; and ABclonal ColorMixed Protein Marker 310 (10–310 kDa), catalog number: RM02943. Blot images, prior to the densitometry readings, were converted to grayscale with ImageJ (ImageJ v.1.49, National Institutes of Health, Maryland, USA) as follows: Image -> Type –> 8 bit, next: Image -> Adjust–> Brightness/Contrast –> Auto.

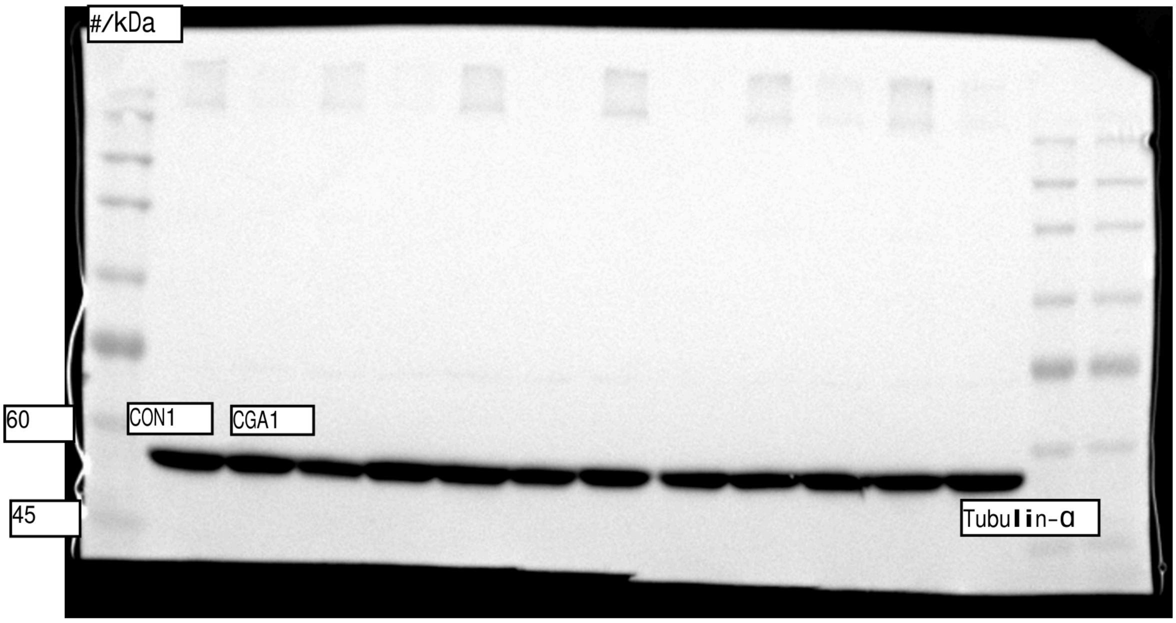

Figure S9. Western blot membrane of Tubulin-α (~55 kDa) protein detected with anti-Tubulin-α (BS1699; 1; 1:10000; Bioworld, USA) antibody. Protein separation was achieved by denaturing polyacrylamide gel electro-phoresis, after which proteins were electrotransferred onto nitrocellulose membranes under constant current conditions. After incubating with the horseradish peroxidase (HRP)-labeled goat anti-rabbit IgG (1:5000), the proteins were determined using the ultra-sensitive ECL chemiluminescence kit (RM00020P, ABclonal, CN) by the digital imaging equipment (GE Healthcare, Chicago, IL, USA). #Weight marker (molecular weight in kDa): ABclonal ColorMixed Protein Marker 180 (10–180 kDa), catalog number: RM19001; and ABclonal ColorMixed Protein Marker 310 (10–310 kDa), catalog number: RM02943. Blot images, prior to the densitometry readings, were converted to grayscale with ImageJ (ImageJ v.1.49, National Institutes of Health, Maryland, USA) as follows: Image -> Type –> 8 bit, next: Image -> Adjust–> Brightness/Contrast –> Auto.
